# A web-based communication system for integrated care in cerebral palsy: experienced contribution to parent-professional communication

**DOI:** 10.5334/ijic.672

**Published:** 2012-03-06

**Authors:** Jitske Gulmans, Miriam Vollenbroek-Hutten, Lisette van Gemert-Pijnen, Wim van Harten

**Affiliations:** Roessingh Research and Development, Institute for Research in Rehabilitation Medicine and Technology, Enschede, The Netherlands; Roessingh Research and Development, Institute for Research in Rehabilitation Medicine and Technology, Enschede, The Netherlands; Faculty of Behavioural Sciences, Department of Psychology and Health and Technology, University of Twente, Enschede, The Netherlands; Department of Health Technology and Services Research, School of Management and Governance, University of Twente, Enschede, The Netherlands

**Keywords:** integrated care, patient care management, cerebral palsy, communication, technology, internet

## Abstract

**Introduction:**

To improve communication in the integrated care setting of children with cerebral palsy, we developed a web-based system for parent-professional and inter-professional communication. The present study aimed to evaluate parents’ experiences regarding the system’s contribution to their communication with professionals during a six-months pilot in three Dutch care regions. In addition, factors associated with parents’ system use and non-use were analyzed.

**Theory and methods:**

The system’s functional specifications were based on key elements of the Chronic Care Model and quality dimensions formulated by the Institute of Medicine. At baseline, parents completed a T0-questionnaire on their experiences regarding sufficiency of contact, accessibility of professionals, timeliness of information exchange, consistency of information and parents’ role as messenger of information and/or care coordinator. After the pilot, parents completed a T1-questionnaire on their experiences regarding the system’s contribution to each of these aspects.

**Results:**

Of the 30 participating parents 21 had used the system, of which 20 completed the T1-questionnaire. All these parents indicated that they had experienced a contribution of the system to parent-professional communication, especially with respect to accessibility of professionals, sufficiency of contact and timeliness of information exchange, and to a lesser extent consistency of information and parents’ messenger/coordinator role. In comparison with non-users, users had less positive baseline experiences with accessibility and a higher number of professionals in the child’s care network.

**Conclusions:**

All users indicated a contribution of the system to parent-professional communication, although the extent of the experienced contribution varied considerably. Based on the differences found between users and non-users, further research might focus on the system’s value for complex care networks and problematic access to professionals.

## Introduction

Children with special health care needs (CSHCN) are an important population from health care services, economic and policy perspectives [1]. The often highly specific and heterogeneous care needs in this population require a broad range of long-term care services from multiple providers across diverse organisations and sectors. A primary aim in such inter-disciplinary and inter-organizational settings is to provide integrated and coordinared care across all elements of the health care system and the patient’s community [2]. This, however, is increasingly difficult to realize given the high level of differentiation (between professionals, units, organisations) [3] and the resulting complexity of health care, characterized by “more to know, more to do, more to manage, more to watch, and more people involved than ever before” [4]. Particularly for the rapidly growing population of chronic patients with multiple concurrent conditions, health care organizations often operate as silos, providing care without complete information about the patient’s condition, medical history or services provided in other settings [4]. In their report *Crossing the quality chasm*, the Institute of Medicine emphasized that health care should be patient-centered, specifying care coordination and integration as one of its key features in order to ensure that accurate information reaches those who need it at the appropriate time [4]. Hereto, effective communication within the health care system and between the health care system and the larger community is of vital importance [4–6] and a fundamental feature in parents’ experiencing services as connected or coordinated [7]. In practice, however, inadequate communication among health care providers and organizations involved in the child’s care is one of the main barriers that challenge care coordination in paediatric services [8]. Based on data of the US National Survey of CSHCN [9], a study among CSHCN populations with neurological conditions found that children with multiple conditions had the greatest unmet needs and dissatisfaction with care coordination, which was defined in terms of communication among doctors and between doctors and other providers and whether the family received sufficient help coordinating care, if needed [10]. Failure of professionals caring for the same child to communicate with one another often leaves the parents as information intermediaries [11] and/or semi-professional care coordinators [7]. This corresponds to our findings on the care of children with cerebral palsy in The Netherlands (Box 1) in which we identified various gaps in communication, such as inadequate cooperation of professionals and an experienced lack of patient-centeredness, as well as insufficient inter-professional contact necessitating parents to take up the role of messenger of information and/or care coordinator [21].

Although much has been written about the potential of eHealth technology to stimulate integrated care across patient conditions, services and sites [22–24], applications in paediatrics have been relatively scarce [25]. More recently, however, they are increasingly being applied to facilitate communication between health care providers and caregivers of paediatric patients with health conditions requiring follow-up [26, 27]. Based on the identified gaps in three Dutch cerebral palsy care regions (Box 2), we aimed to obtain insight in the feasibility and usability of an eHealth application to improve patient care communication in these settings. Hereto, we developed a web-based system for parent-professional and inter-professional communication [31], aimed to increase patient-centeredness, facilitate inter-professional contact and enhance network transparency (see Appendix). Representing an ‘innovator’ phase [32, 33], early prototypes of eHealth technology are generally evaluated on technical stability and user acceptance [34, 35]. As the system proved to be technically feasible in a six-months pilot in three Dutch care regions and most parents reported added value in using the system [31], the present study aimed to evaluate parents’ experiences regarding the system’s contribution to their communication with involved professionals during the six-months pilot. Hereto, we focused on those aspects of parent-professional communication in which we previously identified gaps and hence were targets of improvement for the web-based system, being sufficiency of contact, timeliness of information exchange, accessibility of professionals, consistency of information and the extent to which parents felt they had to act as care coordinator or messenger of information between professionals. In addition, we aimed to gain insight into factors related to parents’ system use and non-use, by comparing parents who had used the system (n=21) with those who had not used the system (n=9) with respect to their baseline experiences with parent-professional communication and the complexity of their child’s care network, hypothesizing that users would have less positive baseline experiences and a more complex care network.

## Theory and methods

### System aims

Following the Chronic Care Model [29], productive interactions between the patient(’s family) and involved practice teams arise from essential system changes at the health care organization level, such as self-management support, delivery system design, decision support and clinical information systems [6]. Although eHealth technology has the potential to contribute to each of these system changes [36], changes with respect to self-management support (how to help patients live with their conditions) and delivery system design (who’s on the health care team and in what ways they interact with patients) will relatively be most visible to the patient(’s family) [6]. As the identified gaps in our study (Box 2) particularly reflected improvement needs in these domains, the primary aims of the web-based system were to increase patient-centeredness, facilitate inter-professional contact and enhance network transparency. In the Appendix, an overview is given of these aims and the corresponding design features. In the present study, we focused on the system’s aim to increase patient-centeredness and hereto evaluated parents’ experiences regarding the system’s contribution to their communication with involved professionals during the six-months pilot.

### Study population

In order to obtain data representative for the integrated care setting of cerebral palsy, three Dutch care regions were included ranging from urban to more rural settings. The selection of patients was carried out by a rehabilitation physician, based on files of patients with annual supervision and the following selection criteria: (1) diagnosis cerebral palsy (confirmed by neurologist) and (2) age between 4–8 years [from the age of four years diagnosis is mostly clear and (special) education becomes an additional communication partner in the care network]. Parents needed to have (1) sufficient Dutch language skills (as judged by the rehabilitation physician) in order to be able to use the communication system and complete questionnaires and (2) access to the internet as well as basic knowledge how to use it. Finally, minimal three involved professionals [i.e. the child’s rehabilitation physician, (primary care) physiotherapist and professional of (special) education/day care centre] needed to participate in the study in order to have a sufficient network for communication. On the basis of these criteria, the parents of 30 cerebral palsy patients were included in the study. After completion of informed consent they received log-in data for access to the system. The study was conducted in keeping with the protocol of the WMA Declaration of Helsinki. According to Dutch legislation (WMO Medical Research Involving Human Subjects Act) a medical ethics review was not required.

### System use and non-use

System use was on a voluntary basis, i.e. parents were free in their choice to use the system in a given situation or apply their usual modes of communication (face-to-face, telephone etc.). The system comprised an open access part (a generally accessible website with project related information) and a personalized secured access part with various consultation options. Parents could contact professionals in their child’s care network, while professionals could contact both parents as well as colleague-professionals involved in the patient’s care network. For each submitted question parents had to mark one feedback-responsible professional, an automatic copy was sent to other involved professionals (parents could remove this option if preferred, see Appendix). Information about parents’ system use was extracted from the system’s database. Of the 30 participating parents, 21 had actually used the system during the six-months pilot by submitting one or more questions and/or responses to professionals, whereas nine parents had only logged into the system without submitting a question/response. Considering the system’s aim to improve parent-professional and inter-professional communication, system use was defined in terms of submitting a question/response on the system more than once. Consequently, trying out the system only once or logging-in without submitting a question/response was not considered actual system use.

### Study design

#### Baseline questionnaire (T0)

In order to describe the study population and to evaluate parents’ experiences with parent-professional communication before the pilot, parents completed a baseline questionnaire, including parent/patient characteristics, frequency and modes of contact with involved professionals in the child’s care network and parents’ experiences regarding their communication with these professionals, focusing on sufficiency of contact, timeliness of information exchange, accessibility of professionals, consistency of information and the extent to which parents felt they had to act as care coordinator and/or messenger of information between professionals. For each of these aspects a scoring table was used in which parents could indicate for each involved professional the extent to which they had experienced sufficient contact/timely information exchange etc. during the preceding six months (3-point Likert scale *‘usually’*, *‘occasionally’* or *‘rarely’*, see Appendix).

#### Questionnaire after six-month pilot (T1)

After the pilot parents completed a questionnaire on the experienced contribution of the system to each of these aspects of parent-professional communication. In addition, parents were asked whether they less often needed face-to-face/telephone contact with involved professionals as a result of using the system. For the items sufficiency of contact, timeliness of information exchange and accessibility of professionals, the experienced contribution was evaluated by means of scoring tables in which parents could indicate for each involved professional the extent to which they had experienced a contribution of the system (3-point Likert scale *‘considerably’*, *‘to some extent’* or *‘(hardly) not’*, see Appendix). For the items consistency of information and the coordinator/messenger role, detailed evaluation per professional was considered less appropriate given the inter-dependency among professionals that is inherent to these aspects of parent-professional communication. Therefore instead of using scoring tables, the experienced contribution for these items was evaluated by means of a 5-point Likert-scale (ranging from *‘yes, definitely’* to *‘no, not at all’*, see Appendix).

#### Data analysis

##### Parents’ questionnaire responses T0/T1

Parents’ T0 questionnaire responses were listed in an overall table, indicating for each parent the proportion of professionals that were scored with a positive/intermediate/negative response (respectively *‘usually’*/*‘occasionally’*/*‘rarely’*, see Table 1). As such, the intermediate and negative responses represented experienced shortcomings in parent-professional communication and thus targets for improvement of the communication system.

Parents’ T1 questionnaire responses on the items sufficiency of contact, timeliness of information exchange and accessibility of professionals were listed in an overall table, indicating parent’s responses regarding the experienced contribution of the system for each involved professional that participated in the study [*‘considerably’*, *‘to some extent’*/*‘(hardly) not’*, see Table 3].

##### Factors associated with parents’ system use and non-use

To evaluate whether parents’ system use was associated with their baseline experiences regarding parent-professional communication, the T0-questionnaire responses of parents who had used the system (use-group, n=21) were compared with the responses of parents who had not used the system (non-use group, n=9), focusing on the proportion of professionals that were scored with a positive experience on the concerning aspect of parent-professional communication. Given the skewed distribution of these proportions, non-parametric tests for independent samples were applied (Mann-Whitney, a=0.05), using one-sided p-values in line with our hypothesis that the use-group would have less positive baseline experiences with parent-professional communication and thus the proportion of professionals that were scored with a positive experience would be lower than in the non-use group. In addition, we evaluated whether the complexity of their child’s care network was associated with parents’ system use, by comparing the amount of involved professionals and institutions between the use and non-use group. For this comparison independent-sample t-tests were applied (a=0.05), using one-sided p-values in line with our hypothesis that the amount of involved professionals and institutions would be higher in the use-group than in the non-use-group.

## Results

### Parents’ baseline experiences (T0)

Table 1 summarises parents’ responses at baseline, both for the parents who had used the system (use, n=21) and the parents who had not used it during the six-month pilot (non-use, n=9). The numbers represent the number of professionals that were marked with a positive (white label), intermediate (grey label) or negative response (black label). As can be seen in the vertical total scores, the majority of professionals were marked with a positive response, although the proportion of positive responses differed between the items, ranging from 88% for consistency of information to 57% for parents as messenger of information. However, looking horizontally at the individual parent level, each parent had scored intermediate or negative responses on one or more items. A relatively high proportion of parents indicated that they had to act as care coordinator or messenger of information: resp. 21 out of 29 parents (72%) and 22 out of 28 parents (79%) had scored one or more professionals with an intermediate and/or negative response. Of the professionals that were marked with a negative response (n=26), the majority were medical specialists (n=14) and paramedical therapists in (special) education-/day care centres (n=8).

### Parents’ system use during the six-month pilot

Of the 30 participating parents, 21 parents had actually used the system, submitting n=111 questions and n=59 responses, with a mean of five questions (range 1–17) and three responses (range 1–9) per parent. As can be seen in Table 2, the rehabilitation physician was most frequently marked as feedback-responsible professional (41% of the 111 submitted questions), next to the physiotherapist and occupational therapist (respectively 20% and 14% of the 111 submitted questions). Overall, (para-)medical professionals were feedback-responsible for the far majority of parents’ questions (90%), whereas education professionals were addressed for feedback in only 10% of the submitted questions.

### Experienced contribution of the system (T1)

Of the 21 parents that had used the system, 20 completed the T1-questionnaire. Table 3 shows their responses regarding the experienced contribution of the system to respectively sufficiency of contact (s), accessibility of professionals (a) and timelinesss of information exchange (t) for each involved professional that participated in the system’s pilot. As can be seen in the overall scores at the bottom of the the table, all 20 parents that completed the T1-questionnaire indicated that for one or more involved professionals the system had to a greater or lesser extent-contributed to sufficient contact, accessibility and/or timely information exchange. In total 14 parents indicated that the system had ‘considerably’ contributed to sufficient contact with one or more involved professionals, particularly the rehabilitation physician (indicated by 10 parents) and the physiotherapist (five parents). With respect to accessibility and timely information exchange, in total 13 parents indicated a considerable contribution of the system, again particularly for the rehabilitation physician (indicated by resp. 8/9 parents) and the physiotherapist (indicated by resp. 9/10 parents). As can be further seen in the table, the number of times that professionals were marked as feedback-responsible for a submitted question (total n=111) was related to the experienced contribution of the system. For those professionals that had not been marked as feedback-responsible for a submitted question (n=0), parents mostly did not experience a contribution of the system. On the other hand, the table shows that even when a professional was only marked once as feedback-responsible for a submitted question (n=1), various parents indicated that they had experienced a considerable contribution of the system for that particular professional.

Considering parents’ responses regarding the experienced contribution of the system to consistency of information and parents’ messenger/coordinator role, less than half of the 20 parents (n=9) indicated a positive response on these items (10%–25% did not know and 25%–35% indicated a negative response).

### Factors associated with parents’ system use and non-use

Comparing the 21 parents who used the system with the nine parents who had not used the system, the non-users scored relatively higher at baseline on accessibility of professionals: 89% of the professionals were marked with a positive experience on this item, compared to 68% for the users, a statistically significant difference (p=0.023). With respect to parents’ baseline experiences regarding sufficiency of contact, both groups scored nearly the same (in the non-use group 75% of the professionals were marked with a positive response on this item, compared to 72% in the use-group). For the remainder of the items, the use-group scored higher than the non-use group, although no significant differences were found.

Comparing the complexity of the child’s care network between both groups, the mean number of involved professionals in the use-group was 8.3 (range 5–14) compared to 5.7 (range 3–10) in the non-use group, a statistically significant difference (p=0.006). The mean number of involved institutions was higher in the use group (mean 4.1, range 2–7) compared to the non-use group (mean 3.7, range 2–5), although this was not a significant difference.

## Discussion

Although the rationale for integrated care is often approached from a system/-organisational perspective in terms of efficiency and cost-effectiveness, the patient-centered imperative and meaning is of increasing importance [37]. In order to improve parent-professional and inter-professional communication in the integrated care setting of children with cerebral palsy, we developed a web-based communication system aimed at increasing patient centeredness, facilitating inter-professional contact and enhancing network transparency. The aim of this study was to evaluate parents’ experiences regarding the system’s contribution to their communication with involved professionals. Based on previous findings [21], the system was expected to contribute to sufficient contact, timely information exchange, accessibility of professionals and consistency of information, as well as to decrease the need for parents to act as care coordinator and messenger of information between professionals. Of the 30 parents that participated in the six-months pilot, 21 had used the system. At baseline, all of them generally experienced good communication with the majority of professionals, but each parent also experienced gaps on one or more aspects, especially sufficiency of contact, accessibility of professionals and the coordinator/messenger role. This corresponds with findings in literature, in which parents reported being the only coordinators of care for their children or the primary method of communication between physicians [7, 11]. Of the 20 users that completed the T1 questionnaire, all had experienced a contribution of the system on one or more aspects. The majority of parents indicated to have experienced a contribution of the system on sufficiency of contact, timely information exchange and accessibility of professionals, whereas consistency of information and the extent to which parents feel care coordinator or messenger of information seemed less influenced by the system. The higher experienced contribution on sufficiency, timeliness and accessibility could be due to a more direct impact of the system on these items, whereas the other aspects might be more dependent on other modes of communication (face-to-face/telephone contact) as well, each affecting consistency of information and the extent to which parents feel care coordinator/messenger of information. Improvement of these aspects might be stimulated through parent-professional discussion of these issues, in which parents are given choices about their role in communicating information between professionals [11]. From a methodological point of view, the differences in experienced contribution could also be due to the fact that the items sufficiency, timeliness and accessibility were assessed by means of a scoring table (in which experienced contribution was operationalized on the level of individual professionals), whereas the system’s contribution to the other items were assessed in more general terms by means of a 5-point Likert scale in which parents could only give overall scores. The choice for a more generic evaluation for these items was made after analysis of parents’ responses in the baseline questionnaire, in which detailed evaluation per professional turned out to be less appropriate considering the inter-dependent nature inherent to these aspects of parent-professional communication.

Although all users had experienced a contribution of the system on one or more aspects, the extent of the experienced contribution varied considerably: some parents experienced a contribution on only one aspect and for just one or two involved professionals, while other parents experienced a contribution on more aspects and for various professionals. This might be partly explained by parents’ differing baseline experiences, but another factor might be the broad variation in frequency of system use (with a mean of n=8 questions/responses per parent, standard deviation 6 and range 2–20). Parents who used the system more frequently might be more likely to have experienced a contribution of the system, although our findings showed that just one submitted question could also positively contribute to parent-professional communication.

Considering the applied methodology to evaluate parents’ experiences, the choice for detailed scoring tables was made in order to evaluate parents’ experiences for each involved professional, and thereby to detect potential disciplines for whom the web-based system might have particular added value. Based on the findings in the present study, this seemed to be the case for the rehabilitation physician and the physiotherapist, whom parents frequently marked as feedback-responsible professionals in submitted questions, and for whom parents experienced a considerable system contribution to sufficient contact, accessibility and/or timely information exchange. However, the reliability of parents’ responses was suboptimal, as could be seen in the total number of marked professionals in the baseline questionnaire item. A more overall quantitative measure or qualitative evaluation might have additional value, although the possibility to detect changes per professional would be lost.

Of the participating parents, almost one-third had not used the system. At baseline, these parents scored significantly higher on accessibility of professionals, which might partly explain their non-use of the system: they already could reach their professionals relatively easily. In these situations of good accessibility of professionals, a web-based system might therefore be less indicated. In line with this, we found that the complexity of the care network (measured by means of the amount of involved professionals and institutions) was higher in the use-group than in the non-use group. Based on the differences found between the use- and non-use group, we hypothesize that the system may be especially valuable in patient populations with complex care networks involving multiple professionals and institutions, and less positive experiences with accessibility of professionals.

## Conclusions

All users experienced a contribution of the system to parent-professional communication, although the extent of the experienced contribution varied considerably. The strength of the system appears to lie in its contribution to sufficient contact, timely information exchange and accessibility of professionals, whereas consistency of information and the coordinator/messenger role seemed less influenced by the system. In line with a staged approach of telemedicine evaluation, these findings can be taken into account in the further development of the system, ranging from optimization of the system by expanding consultation possibilities and providing insight into the consultation process, to a more specific definition of the system’s target population, focusing on patient populations with complex care networks and problematic access to professionals. In addition, innovative methods such as social network analysis might be applied to gain insight into the strength of parent-professional and inter-professional relationships as a proxy for success in integrated care [38].

## Figures and Tables

**Box 1.** Integrated care for children with cerebral palsy

Cerebral palsy is one of the most severe chronic disabilities in childhood, often making strong demands on health, education and social services as well as on families and children themselves [12]. In The Netherlands, children with cerebral palsy are the largest diagnostic group treated in paediatric rehabilitation [13], with a prevalence ranging from 1.5 to 2.5 per 1000 live births with little or no variation among Western nations [14, 15]. Cerebral palsy has usually been defined as an umbrella term covering a group of motor disorders caused by a non-progressive lesion of the immature brain [16]. More recently, activity limitation was added as conditional feature and an annotation was made that the motor disorders are often accompanied by disturbances of sensation, perception, cognition, communication, and behaviour, by epilepsy, and by secondary musculoskeletal problems [17]. As no two children are affected in the same way, individual treatment programs vary widely, presenting care providers with heterogeneous and complex diagnostic and therapeutic challenges, requiring a broad range of specialized services from various professionals across diverse institutions and settings [18]. Although one of the primary aims in such interdisciplinary and -organizational settings is to provide integrated care, a study on integrated paediatric services in The Netherlands concluded that despite the fact that family-centered and coordinated care are seen as the two most desirable and effective ways of paediatric care delivery, their practical implementation in Dutch paediatric practice is still in a preliminary stage [19]. In line with this, a descriptive quality inventory of cerebral palsy care in The Netherlands identified suboptimal communication across institutions and settings as one of the main gaps in care coordination [20]. In view of these challenges, the overall aim of our study is to contribute to the improvement of patient care communication across the integrated care setting of cerebral palsy in three Dutch care regions.

**Box 2.** Improving communication in cerebral palsy care

To identify experienced gaps in communication across the integrated care setting of cerebral palsy, we searched the literature for appropriate research methodology. Existing methods though were often restricted to only one aspect of communication (e.g. discharge- or referral communication), one communication link (e.g. general practitioner–hospital specialist) or one evaluation perspective (e.g. the perspective of primary care physicians), or relied solely on quantitative-resp. qualitative methods, thus obtaining either general/population based data or in-depth qualitative data derived from small samples [28]. In view of these shortcomings, we developed a mixed method evaluation approach [28], based on key elements of the Chronic Care Model [6, 29], quality of care aspects formulated by the Institute of Medicine [4] and essential quality dimensions of information(-exchange) [30]. Application of this approach in three Dutch cerebral palsy care regions [21] showed that parents primarily experienced gaps in inter-professional communication, particularly between the (rehabilitation) hospital and primary care physiotherapy resp. (special) education/day care centre. Subsequent in-depth interviews with a subset of parents showed that the experienced gaps were primarily related to inadequate cooperation of professionals and an experienced lack of patient-centeredness, as well as insufficient inter-professional information-exchange and consistency of information, which often necessitated parents to take up the role of messenger of information or even that of care coordinator [21]. Confronting professionals with these findings yielded further understanding of underlying factors, such as capacity problems and a lack of interdisciplinary guidelines and clear definition of roles, tasks and responsibilities [21]. Based on these gaps in communication, we developed an asynchronous secure web-based system for parent-professional and inter-professional communication, aimed at increasing patient centeredness, facilitating inter-professional contact and enhancing network transparency [31]. For each of these aims, functional specifications were formulated, which were subsequently translated into technical requirements (see Appendix). Based on the findings of a six-month pilot-evaluation in three Dutch care regions, the system proved to be technically robust and reliable [31]. Approximately two-thirds of the parents and half of the professionals had used the system, of which most parents and some professionals reported to have experienced added value in its use [31], comprising each of the three system aims: patient-centeredness (parents could ask questions at the moment they arose and the whole network could be reached at once, avoiding fruitless phone calls), inter-professional contact (lower threshold for consultation, contact with disciplines which previously were not actively involved in decision making) and network transparency (professionals were being kept up to date between visits, obtaining insight about other professionals’ advice; parents could re-view their communication with professionals) [31].

**Table 1.**
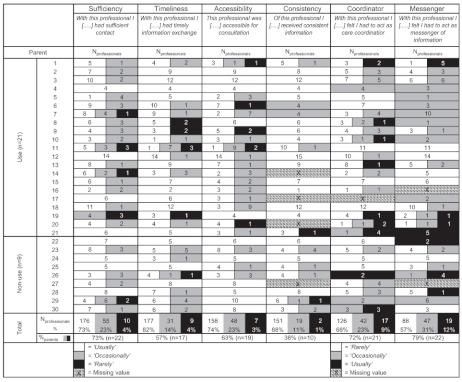
Parents’ baseline experiences regarding parent-professional communication

**Table 2.** Overview of feedback-responsible professionals in parents’ submitted questions (n=111).

			Questions	
			n	%
Care region		Region A (urban)	34	31
		Region B (urban/rural)	16	14
		Region C (rural)	61	55
Institution		Hospital	27	24
		Rehabilitation centre	48	43
		(Special) education/day care centre	19	17
		Primary care centre	17	15
Discipline		Medical	49	44
		Paramedical	51	46
		Educational	11	10
	Medical	Rehabilitation physician	45	41
		Paediatrician	3	3
		Paediatric neurologist	1	1
	Paramedical	Physiotherapist	22	20
		Occupational therapist	15	14
		Manufacturer rehabilitation aids	5	5
		Speech therapist	2	2
		Social work	2	2
		Orthoptist	2	2
		Pedagogue	1	1
		Dietician	1	1
		Creative therapist	1	1
	Educational	Teacher	8	7
		(Ambulant) supervisor	2	2
		Group leader (day care)	1	1

**Table 3.**
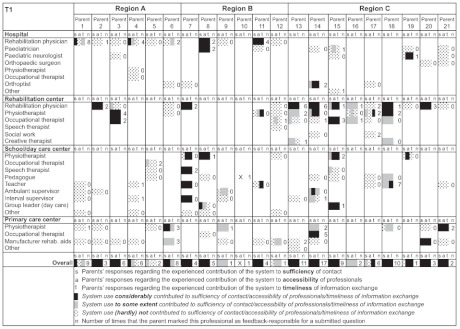
Parents’ responses regarding the experienced contribution of the system to sufficiency of contact **(s)**, accessibility of professionals **(a)** and timelinesss of information exchange **(t)**

**Appendix Table X1.**
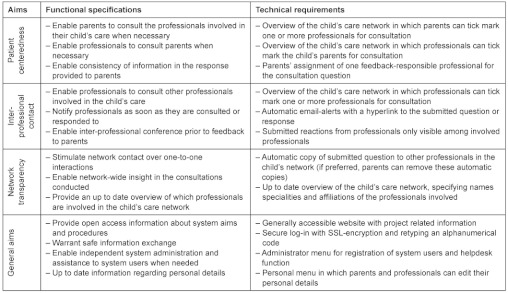
System aims and corresponding functional specifications and technical requirements [31]

**Table X2.**
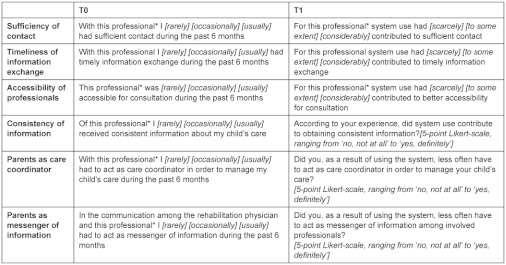
Questionnaire items at baseline (T0) and after the pilot (T1). Items marked with * were assessed by means of a scoring table in which parents could indicate their response for each involved professional

## Reviewers

**Stephen J. Gentles**, MSc, McMaster University, Department of Clinical Epidemiology & Biostatistics, 1280 Main St W, Hamilton, ON, L8S 4K1, Canada

**K. Ann McKibbon**, PhD, Associate Professor, McMaster University, Department of Clinical Epidemiology & Biostatistics, 1280 Main St W, Hamilton, ON, L8S 4K1, Canada

**Ingrid Mur-Veeman**, Department of HSR, Maastricht University, School for Public Health and Primary Care (CAPHRI), Faculty of Health, Medicine and Life Sciences, P.O. Box 616, 6200 MD Maastricht, Netherlands

**Christopher Stille**, MD, MPH, Professor and Head, General Academic Pediatrics, University of Colorado Denver/Children’s, Hospital Colorado, 13123 East 16th Avenue, B032, Aurora, CO 80045, USA

One anonymous reviewer.

## References

[1] Van Dyck PC, Kogan MD, McPherson MG, Weissman GR, Newacheck PW (2004). Prevalence and characteristics of children with special health care needs. Archives of Pediatrics and Adolescent Medicine.

[2] AAP, AAFP, ACP, and AOA (2007). Joint principles of the patient-centered medical home. Delaware Medical Journal.

[3] Van Wijngaarden JDH, De Bont AA, Huijsman R (2006). Learning to cross boundaries: the integration of a health network to deliver seamless care. Health Policy: Education, Health Service Delivery and Regulation.

[4] Institute of Medicine (2001). Crossing the quality chasm: a new health system for the 21st century.

[5] Stille CJ, Antonelli RC (2004). Coordination of care for children with special health care needs. Current Opinion in Pediatrics.

[6] Wagner EH, Bennett SM, Austin BT, Greene SM, Schaefer JK, Vonkorff M (2005). Finding common ground: patient-centeredness and evidence-based chronic illness care. Journal of Alternative and Complementary Medicine.

[7] Miller AR, Condin CJ, McKellin WH, Shaw N, Klassen AF, Sheps S (2009). Continuity of care for children with complex chronic health conditions: parents’ perspectives. BioMed Central Health Services Research.

[8] American Academy of Pediatrics (2005). Council on Children with Disabilities. Care coordination in the medical home: integrating health and related systems of care for children with special health care needs. Pediatrics.

[9] Kogan MD, Strickland BB, Newacheck PW (2009). Building systems of care: findings from the national survey of children with special health care needs. Pediatrics.

[10] Bitsko RH, Visser SN, Schieve LA, Ross DS, Thurman DJ, Perou R (2009). Unmet health care needs among CSHCN with neurologic conditions. Pediatrics.

[11] Stille CJ, Primack WA, McLaughlin TJ, Wasserman RC (2007). Parents as information intermediaries between primary care and specialty physicians. Pediatrics.

[12] Surveillance of Cerebral Palsy in Europe (SCPE) (2000). Surveillance of cerebral palsy in Europe: a collaboration of cerebral palsy surveys and registers. Developmental Medicine and Child Neurology.

[13] Odding E, Roebroeck ME, Stam HJ (2006). The epidemiology of cerebral palsy: incidence, impairments and risk factors. Disability and Rehabilitation.

[14] Paneth N, Hong T, Korzeniewski S (2006). The descriptive epidemiology of cerebral palsy. Clinics in Perinatology.

[15] Wichers MJ, Van der Schouw YT, Moons KG, Stam HJ, Van Nieuwenhuizen O (2001). Prevalence of cerebral palsy in The Netherlands (1977–1988). European Journal of Epidemiology.

[16] Mutch L, Alberman E, Hagberg B, Kodama K, Perat MV (1992). Cerebral palsy epidemiology: where are we now and where are we going?. Developmental Medicine and Child Neurology.

[17] Rosenbaum P, Paneth N, Leviton A, Goldstein M, Bax M, Damiano D (2007). A report: the definition and classification of cerebral palsy April 2006. Developmental Medicine and Child Neurology. Supplement.

[18] Cooley WC (2004). Providing a primary care medical home for children and youth with cerebral palsy. Pediatrics.

[19] Nijhuis BJ, Reinders-Messelink HA, De Blécourt AC, Olijve WG, Haga N, Groothoff JW (2007). Towards integrated paediatric services in the Netherlands: a survey of views and policies on collaboration in the care for children with cerebral palsy. Child: Care, Health and Development.

[20] Van der Salm A, Van Harten WH, Maathuis CGB (2001). Quality of the cerebral palsy care chain—A qualitative inventory of Dutch cerebal palsy care and its potential improvements [in Dutch].

[21] Gulmans J, Vollenbroek-Hutten MMR, Van Gemert-Pijnen JEWC, Van Harten WH (2009). Evaluating patient care communication in integrated care settings: application of a mixed method approach in cerebral palsy programs. International Journal for Quality in Health Care.

[22] Institute of Medicine (2011). Health IT and patient safety: building safer systems for better care.

[23] Nijland N (2011). Grounding eHealth: towards a holistic framework for sustainable eHealth technologies..

[24] World Health Organization (WHO) (2010). How can telehealth help in the provision of integrated care?.

[25] Spooner SA, Gotlieb EM (2004). Telemedicine: pediatric applications. Pediatrics.

[26] Gentles SJ, Lokker C, McKibbon KA (2010). Health information technology to facilitate communication involving health care providers, caregivers, and pediatric patients: a scoping review. Journal of Medical Internet Research.

[27] McConnochie KM (2006). Potential of telemedicine in pediatric primary care. Pediatrics in Review.

[28] Gulmans J, Vollenbroek-Hutten MMR, Van Gemert-Pijnen JEWC, Van Harten WH (2007). Evaluating quality of patient care communication in integrated care settings: a mixed method approach. International Journal for Quality in Health Care.

[29] Wagner EH (1998). Chronic disease management: what will it take to improve care for chronic illness?. Effective Clinical Practice.

[30] Lee YW, Strong DM, Kahn BK, Wang RY (2002). AIMQ: a methodology for information quality assessment. Information and Management: The International Journal of Management Processes and Systems.

[31] Gulmans J, Vollenbroek-Hutten MMR, Visser JJW, Oude Nijeweme-d’Hollosy W, Van Gemert-Pijnen JEWC, Van Harten WH (2010). A web-based communication system for integrated care in cerebral palsy: design features, technical feasibility and usability. Journal of Telemedicine and Telecare.

[32] Cain M, Mittman R (2002). Diffusion of innovation in health care.

[33] Rogers EM (2004). Diffusion of innovations.

[34] Broens TH, Huis in’t Veld RM, Vollenbroek-Hutten MMR, Hermens HJ, Van Halteren AT, Nieuwenhuis LJ (2007). Determinants of successful telemedicine implementations: a literature study. Journal of Telemedicine and Telecare.

[35] DeChant HK, Tohme WG, Mun SK, Hayes WS, Schulman KA (1996). Health systems evaluation of telemedicine: a staged approach. Telemedicine Journal: The Official Journal of the American Telemedicine Association.

[36] Wiecha J, Pollard T (2004). The interdisciplinary eHealth team: chronic care for the future. Journal of Medical Internet Research.

[37] Kodner DL, Spreeuwenberg C (2002). Integrated care: meaning, logic, applications, and implications—a discussion paper. International Journal of Integrated Care [serial online].

[38] Goodwin N (2010). It’s good to talk: social network analysis as a method for judging the strength of integrated care. International Journal of Integrated Care [serial online].

